# Influence of Probing System Configuration and Sampling Strategy on Roundness and Cylindricity Measurements Using a Coordinate Measuring Machine

**DOI:** 10.3390/ma19214721

**Published:** 2026-11-06

**Authors:** Dana Kubátová, Václav Blábol, Kateřina Skovajsa, Jakub Čása

**Affiliations:** Department of Machining Technology, Faculty of Mechanical Engineering, University of West Bohemia in Pilsen, 30100 Pilsen, Czech Republic; vblabol@fst.zcu.cz (V.B.); kasovak@fst.zcu.cz (K.S.);

**Keywords:** coordinate measuring machine, CMM, coordinate metrology, roundness, cylindricity, probing system, stylus length, probing ball, sampling density, ANOVA

## Abstract

Coordinate measuring machines (CMMs) report sampling-dependent estimates of geometric form. This secondary, exploratory reanalysis examines individual exported results from six drilled cylindrical features documented in a 2025 diploma thesis, with two probing heads and sampling levels of 5, 20 and 100 points per circular section. Historical ANOVA summaries, a Holm sensitivity analysis and exploratory Kruskal–Wallis comparisons consistently identify sampling density as the most recurrent association with the evaluated roundness and cylindricity. These statistical results are not confirmatory: the reduced, partly confounded design lacks independent replications of exact measurement configurations, while repeated observations concern the same physical features. Newly consolidated absolute and relative contrasts quantify the scale and feature dependence of the reported changes, and machine-readable data expose the underlying configurations and documentation limitations. Comparison with Talyrond reference results remains descriptive because equivalence of evaluation operators and a combined task-specific uncertainty have not been established. The contribution is therefore a transparent, reproducible reassessment of how inspection choices affect reported CMM form values, not an independent causal demonstration or proof of improved measurement accuracy.

## 1. Introduction

Coordinate measuring machines (CMMs) are widely used for dimensional and geometrical inspection because they allow multiple geometrical characteristics to be evaluated on a single measurement platform [1,2]. However, task-specific measurement performance depends not only on machine accuracy but also on the probing system, measurement strategy, feature geometry, environmental conditions, and the mathematical evaluation of measured coordinates [1,3].

This distinction is particularly important in form metrology. Roundness and cylindricity are reconstructed from a finite set of coordinates representing the actual surface; consequently, the evaluated deviation may depend on both the density and spatial distribution of the acquired points. Cylindricity terminology and specification operators are defined within the ISO GPS framework [4,5], while ISO 10360-5:2020 addresses CMMs using single and multiple stylus contacting probing systems in discrete-point and scanning modes [6].

The probing system forms the physical interface between the CMM and the workpiece. Ali [7] showed that probe-related characteristics can contribute to coordinate-measurement error. Mazur et al. [8] subsequently demonstrated that probe orientation and swivel length can affect contact-point identification. Stylus length is therefore a relevant task-specific parameter; Parthiban et al. [9] reported measurable differences in CMM roundness results obtained with different probe lengths.

Sampling strategy is particularly important for form evaluation. Stępień et al. [10] investigated roundness measurements using a ZEISS Prismo with a VAST GOLD probe and demonstrated that measurement conditions and sampling can affect evaluated roundness. Liu et al. [11] likewise highlighted the importance of large point datasets in minimum-zone cylindricity evaluation. More generally, Štrbac et al. [12] emphasized the role of measurement-strategy selection in CMM inspection, while Sato et al. [13] showed how experimental design and ANOVA can support task-specific uncertainty evaluation.

The objective is to conduct a documented secondary, explicitly exploratory analysis of the associations between probing-head type, ball diameter, stylus configuration, sampling density and CMM-reported form values for six drilled features. The expected sampling effect is well known and is not claimed as a new physical phenomenon. Instead, the specific contribution is to (i) quantify feature-to-feature variation using common absolute and relative contrasts; (ii) assess the sensitivity of historical significance classifications to multiple-testing adjustment and a separately labelled exploratory nonparametric analysis; (iii) make observation-level exported results and their provenance accessible; and (iv) identify design and reference-comparison limitations that condition their industrial interpretation. This study does not estimate independently identifiable causal factor effects or establish a general optimal sampling density.

The source measurements were documented in a diploma thesis completed in 2025 [14], only one year before preparation of this 2026 manuscript; the source thesis is therefore recent rather than an old publication, although the thesis date alone does not establish the dates of individual measurements. The publication contribution is nevertheless distinct from data recency: it consists of a harmonized overview of six cylindricity and eighteen roundness outputs; new across-diameter absolute and percentage contrasts calculated from published means; a methodological audit of the reduced, incompletely replicated design and inherited ANOVA; and task-oriented guidance on interpreting sampling and probe-configuration effects. These descriptive derived contrasts are new to the present presentation, whereas the measurements and original significance tests are not.

## 2. Materials and Methods

The experimental program was carried out and documented in Čása’s 2025 diploma thesis [14]. This 2026 article is a secondary analysis and critical synthesis of the experimental record documented in 2025, not a report of newly collected CMM observations. Specifically, the reworking combines the previously separate results across six feature sizes and eighteen axial-section datasets, adds comparable changes expressed in micrometres and percent of the sparsely sampled estimate, scrutinizes the underlying experimental limitations, and formulates industrial interpretation criteria. This separation of source experiment and present analytical contribution is essential for reproducibility and a fair appraisal of novelty.

### 2.1. Coordinate Measuring Machine and Experimental Conditions

**Figure 1:**
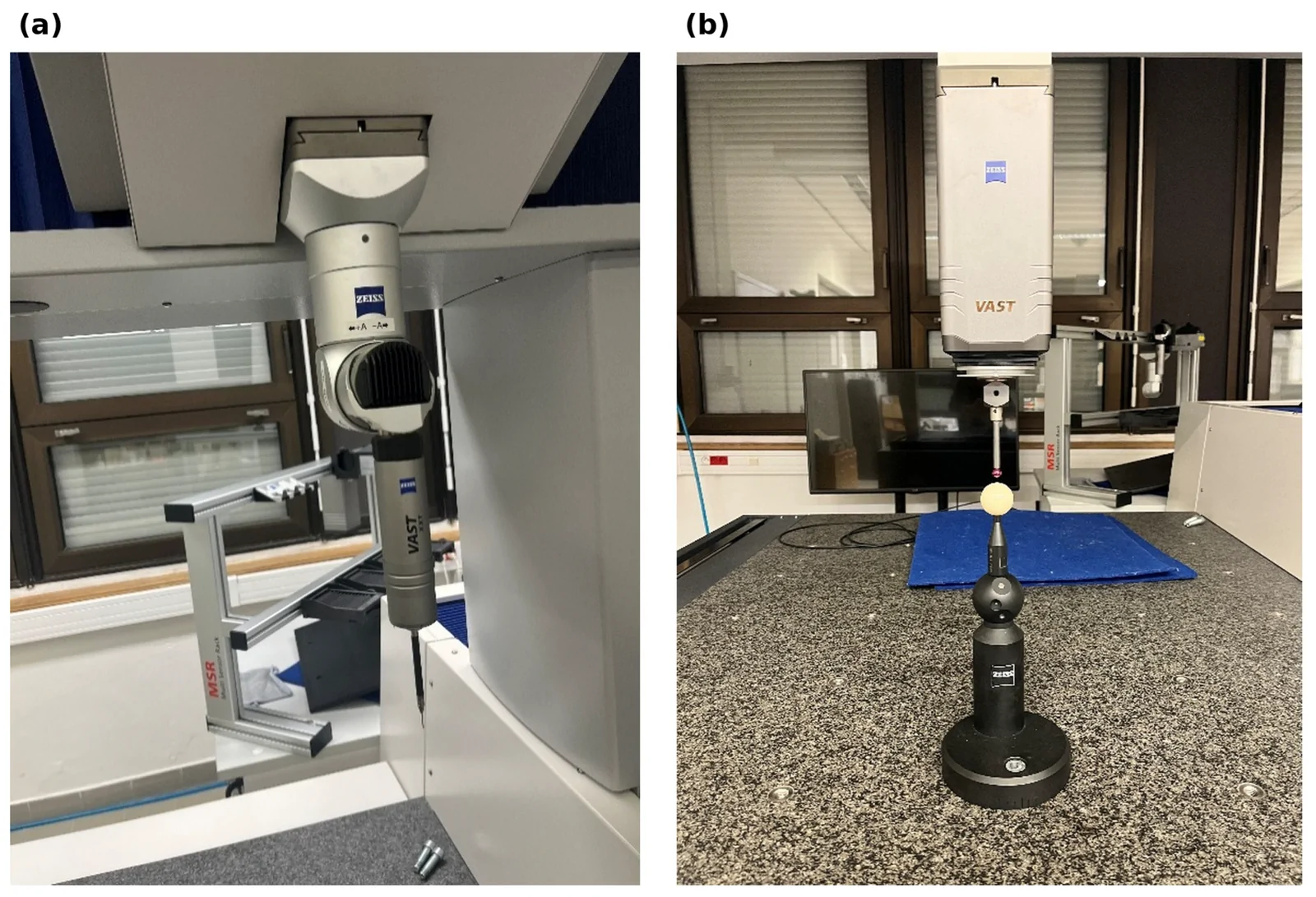
Contact-probing systems used in the experiment: (**a**) articulating ZEISS VAST XXT and (**b**) fixed ZEISS VAST GOLD. Original photographs from the experimental work.

Measurements were performed on a ZEISS PRISMO 7 Navigator (Carl Zeiss—ZEISS Industrial Metrology, Oberkochen, Germany) coordinate measuring machine using ZEISS CALYPSO 2021 software (Carl Zeiss—ZEISS Industrial Metrology, Germany). Two contact-probing configurations were investigated: the fixed ZEISS VAST GOLD head and the articulating ZEISS VAST XXT head. The instrument performance specifications reported for these configurations were (0.9 + L/350) µm for VAST GOLD (Carl Zeiss—ZEISS Industrial Metrology, Germany) and (1.6 + L/350) µm for VAST XXT (Carl Zeiss—ZEISS Industrial Metrology, Germany), where L is the measured length in metres. These are configuration-specific instrument specifications, not uncertainty estimates for the roundness or cylindricity tasks. The laboratory temperature ranged from 20.2 to 20.6 °C and relative humidity from 44% to 50%. The two probing heads are shown in Figure 1.

### 2.2. Reference Form Measurements

Independent reference measurements were performed using a Taylor Hobson Talyrond 585 LT (AMETEK—Taylor Hobson, Leicester, UK) form-measuring instrument. Reference cylindricity values are summarized in Table 1. Roundness was evaluated at three nominal axial positions for each feature; however, the original Talyrond and CMM exports do not consistently identify the third D19.8 section with the same depth label. Consequently, exact axial correspondence is not assumed for that reference comparison (Section 2.3).

For the Taylor Hobson Talyrond 585 LT, the instrument accuracy stated for this study is 0.001 µm, and the reported reference-instrument measurement uncertainty is 0.1 µm (both values provided by the authors). These are distinct metrological quantities and should not be conflated. The 0.1 µm value is the expanded uncertainty U with coverage factor k = 2, corresponding to u = U/k = 0.05 µm; the associated measurement conditions have not been fully documented. Accordingly, the stated reference-instrument uncertainty is not used as a complete uncertainty budget or an acceptance limit for differences between instruments. The available records also do not establish the Talyrond acquisition point count, geometric fitting operator or filter settings. The reference values are therefore used for descriptive comparisons rather than uncertainty-normalized conformity assessment.

For the ZEISS PRISMO 7 Navigator, the specified values differ between the two installed heads. Consequently, the same generic CMM accuracy expression must not be assigned to both head configurations. The preliminary ± 0.001 mm threshold is retained only as a historical screening rule; it is not derived from either configuration-dependent specification or from the Talyrond accuracy figure.

The source thesis (Čása [14], Section 3.7.1, Tables 3-5 and 3-6) records the Talyrond reference values and separately quotes an LSCI-related performance expression of 0.015 + 0.00025 µm/mm. Its specification context is not sufficiently established to reconcile it with the separately reported accuracy (0.001 µm) and uncertainty (0.1 µm); these figures are therefore presented separately. The expanded uncertainty U = 0.1 µm (k = 2) pertains to the reference instrument and does not quantify the full task-specific uncertainty of the CMM–Talyrond difference, which also depends on CMM measurement effects and differing evaluation strategies. The screening comparison is presented in Table 2.

### 2.3. Measured Features and Axial Positions

**Figure 2:**
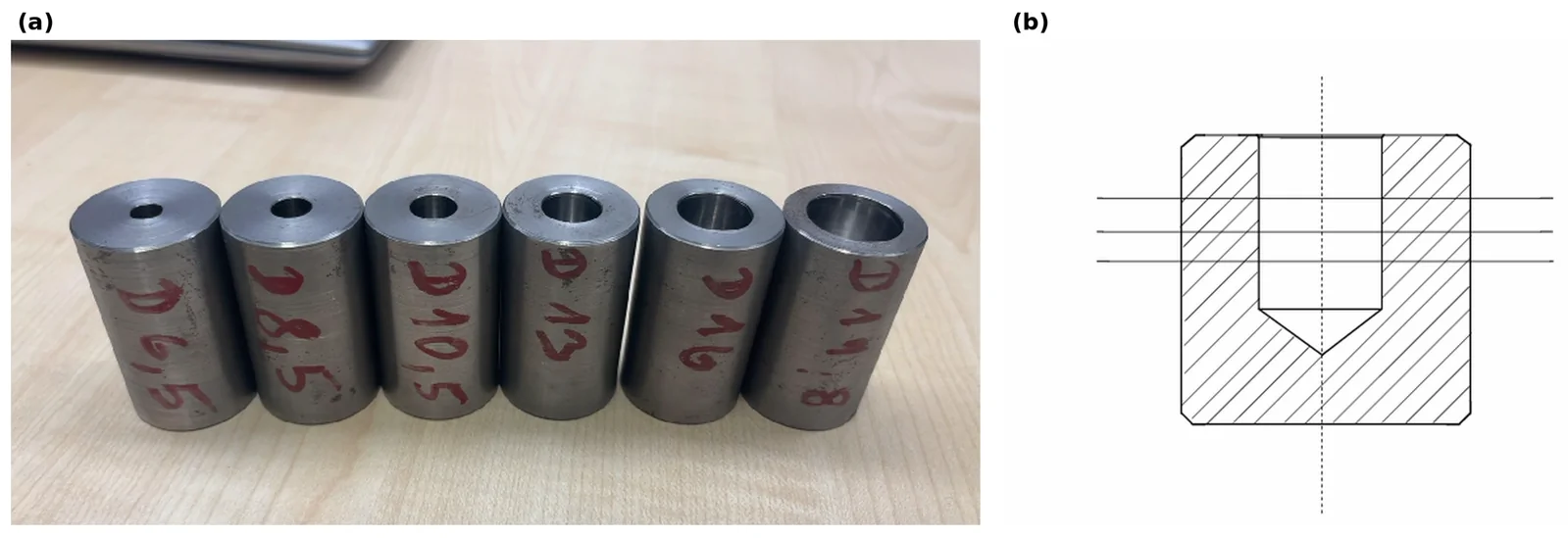
Experimental geometry: (**a**) cylindrical specimens with drilled holes used in the study and (**b**) distribution of the three axial measurement sections used for form evaluation. Original material from the experimental work.

Six drilled cylindrical features with nominal diameters of 6.5, 8.5, 10.5, 13, 16, and 19.8 mm were investigated. Each feature was evaluated at three axial positions selected to cover the accessible measurement depth. Figure 2 shows the investigated specimens and the original measurement-section arrangement used in the experimental design.

The documented dataset contains six nominal feature sizes and three investigated sections per feature, Table 3. The source material reproduced here does not establish independent replication across separately manufactured features of the same nominal diameter, nor does it provide verified material grade, surface-finish parameters or machining conditions beyond drilling. Accordingly, the conclusions apply to the measured features and must not be extrapolated to material classes or production populations.

The thesis describes one drilled cylindrical test piece for each of the six nominal hole diameters, selected at common drilling depth, with the holes cleaned using solvent and compressed air before thermal stabilization in the laboratory (thesis Section 3.1 and Section 3.2). Material grade and numerical surface roughness are not specified in the cited experimental description.

### 2.4. Probing-Ball Diameter and Stylus Length

Three probing-ball diameters were selected for each feature size, sea Table 4. Multiple stylus lengths were then combined with the selected ball diameters according to accessibility. For example, D19.8 used 3, 5, and 8 mm balls with stylus-length sets of 25/35/50 mm, 50/75/130 mm, and 63/110/175 mm, respectively.

Probing-ball diameters and stylus lengths were selected as feasible configurations subject to feature accessibility. They do not form a fully crossed design: available shaft lengths depend on the ball diameter and feature size. Thus, a measured stylus or ball association may partially reflect the distribution of other configuration parameters, and individual causal effects cannot be isolated reliably. Manufacturer-based selection criteria beyond the available configuration record cannot be verified.

According to the source thesis (Čása [14], Section 3.4, Tables 3-1 and 3-2), the fixed and articulating systems used M5 and M3 stylus mounts, respectively. Stylus lengths were chosen to be close but could not always be identical because of commercially available configurations and cost. This introduces structural dependence between probe-head type, stylus length and probe-ball diameter; thus, their coefficients should not be read as independently randomized causal effects.

The three axial positions were selected with regard to probing accessibility and available stylus length (Čása [14], Section 3-7). The CMM roundness and three-section cylindricity analysis used nominal depths of 1.5/3/4 mm (D6.5 and D8.5), 2/3/4 mm (D10.5), 3/5/8 mm (D13 and D16), and 5/12/13 mm (D19.8). Diameter and roundness were acquired using separate measurement strategies and evaluated separately in ZEISS CALYPSO, as confirmed by the experimental investigators. Accordingly, their exported axial labels must not be equated without independent verification. For D19.8, both probing-head exports identify the final roundness characteristic as h13 and the final diameter characteristic as h17, whereas the Talyrond reference export labels the final roundness result h15. The available record does not establish that the Talyrond h15 and CMM h13 results represent the same physical cross-section. This reference pair was therefore excluded from depth-matched roundness agreement calculations; the original labels and numerical observations are retained in Supplementary Dataset S1. These axial positions are not independent replicate specimens.

### 2.5. Sampling Strategy

Roundness was measured using 5, 20, and 100 points per circular section. Cylindricity was evaluated from three circular sections, corresponding to total point counts of 15, 60, and 300. These levels were used consistently across the nominal diameters.

The three sampling levels represent an intentionally broad sparse-to-denser discrete-point comparison, rather than a validated prescription of industrial point counts. Five points provide a coarse profile representation; 20 points offer an intermediate strategy; and 100 points increase the probability of encountering local extrema at the cost of additional acquisition time. Their appropriateness for a particular tolerance, spatial frequency or industrial cycle time was not independently established. For cylindricity, the corresponding counts are 15, 60 and 300 across three sections. The source report does not document angular offsets, point-distribution regularity or whether 100 points yield convergence to a stable form estimate.

The thesis (Čása [14], Section 3.7) explains the selection of 5, 20 and 100 points in terms of the minimum three points required to define a circle, previous measurement experience and stylus service life. The use of 20 points as a suitable production compromise remains a hypothesis; acquisition-time data and convergence measurements are not reported.

The measurements were programmed in ZEISS CALYPSO 2021. Diameter and roundness were acquired under distinct measurement strategies and evaluated as separate characteristics. The source thesis documents the use of three circular sections for the reported cylindricity evaluation and describes specimen alignment using the end face and a hole circle (Čása [14], Section 3.5). Although least-squares and minimum-zone operators are reviewed theoretically in the thesis, the exact saved fitting operators and filtering settings for each characteristic are not unambiguously documented. No equivalence of the CMM and Talyrond fitting/filter operators is therefore assumed.

### 2.6. Preliminary Reference Comparison

Before the broader multifactor evaluation, an initial comparison was performed using the three probing-ball diameters assigned to each cylindrical feature, the medium stylus shaft length, and 10 measured points. Five repeated measurements were performed with each probing-ball diameter. Results obtained with the fixed VAST GOLD and articulating VAST XXT heads were compared with the Talyrond reference values. For this preliminary comparison, the diploma thesis used ±0.001 mm as a working comparison criterion; this criterion is reported here only to reproduce the original evaluation and is not interpreted as a formal measurement-uncertainty interval.

The preliminary 10-point protocol is a separate, repeated screening comparison, not one of the 5/20/100-point levels used in the subsequent multifactor experiment. Because the two protocols differ in sampling density, the preliminary percentages are not directly interchangeable with the later statistical effects. The origin of the ±0.001 mm working threshold is not documented as an instrument uncertainty, specification tolerance or acceptance limit; it should therefore be understood only as a historical descriptive threshold, not evidence of compliance or accuracy. The reported Talyrond uncertainty of 0.1 µm does not validate the historical ±1 µm screening criterion, since the latter is not a combined uncertainty of the measurement difference.

The original preliminary comparison consisted of five repetitions at each ball diameter with a medium stylus length and 10 points (Čása [14], Section 3.7.3). The source thesis associates the ±0.001 mm comparison threshold with the reported CMM performance, but this is not equivalent to a calibrated uncertainty or a feature-specific form tolerance. Accordingly, it is used in the present article solely as an informal descriptive criterion.

### 2.7. Statistical Analysis

The historical diploma-thesis analysis reported descriptive statistics and factorial ANOVA at a nominal α = 0.05 for ball diameter, stylus length, sampling density and probing-head type, followed in selected cases by least significant difference (LSD) comparisons. These original model outputs are reproduced solely as exploratory summaries of the observed reduced design. Because observations are repeated on the same physical features, several factor levels are structurally linked and exact configurations lack independent replication, the classical model assumptions and independent-factor effects are not established. Accordingly, nominal F- and *p*-values describe patterns in the archived data under the historical fitted model; they are not confirmatory tests, estimates of causal effects or independent metrological validation.

The thesis reports historical ANOVA model summaries, including factor and residual degrees of freedom for individual datasets (e.g., residual df = 27 for D10.5 cylindricity and 28 for D6.5 cylindricity). However, a fully verified observation-level design matrix and residual diagnostics are not reproduced for every feature. Residual normality, homogeneity of variance, measurement dependence and the identifiability of all relevant interactions cannot be verified systematically from those summaries. The reported ANOVA is therefore retained as an exploratory historical analysis, rather than newly validated independent-factor inference.

**Figure 3:**
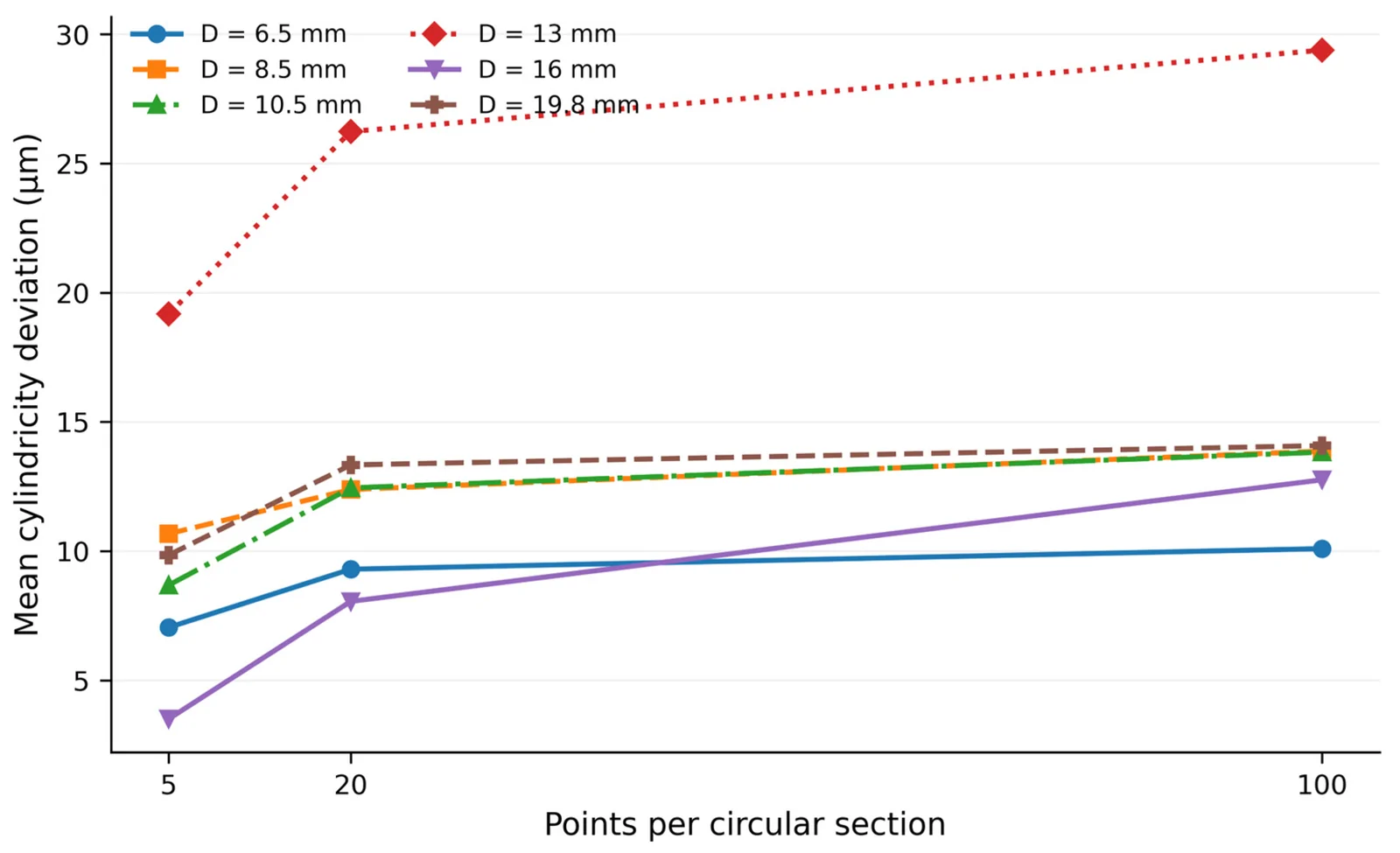
Mean cylindricity deviation as a function of sampling density for all investigated nominal diameters. Cylindricity was evaluated from three axial sections; the x-axis reports points per circular section (5, 20, and 100), corresponding to total point counts of 15, 60, and 300.

**Figure 4:**
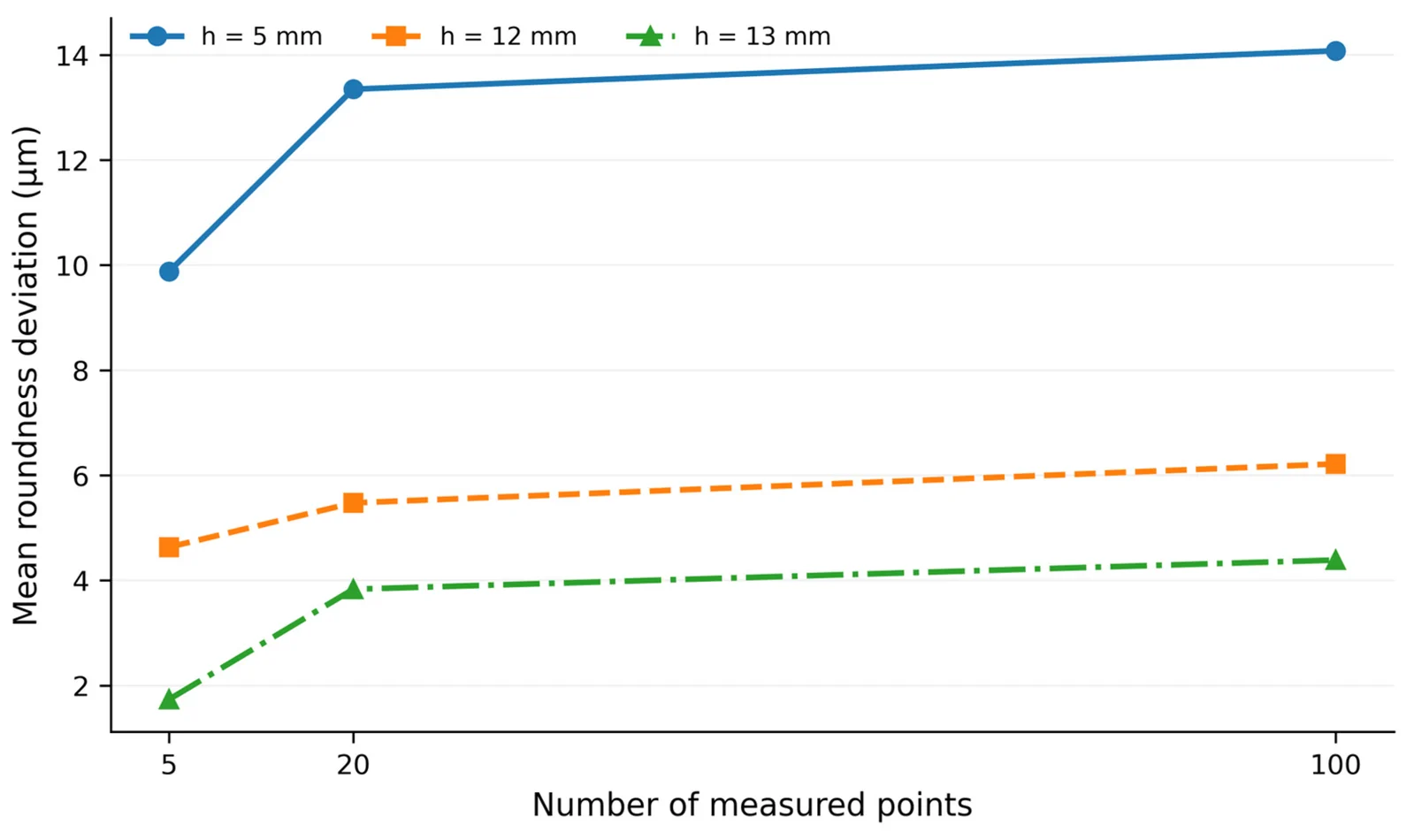
Effect of the number of measured points on mean roundness deviation for the D19.8 mm feature at h = 5, 12, and 13 mm.

**Figure 5:**
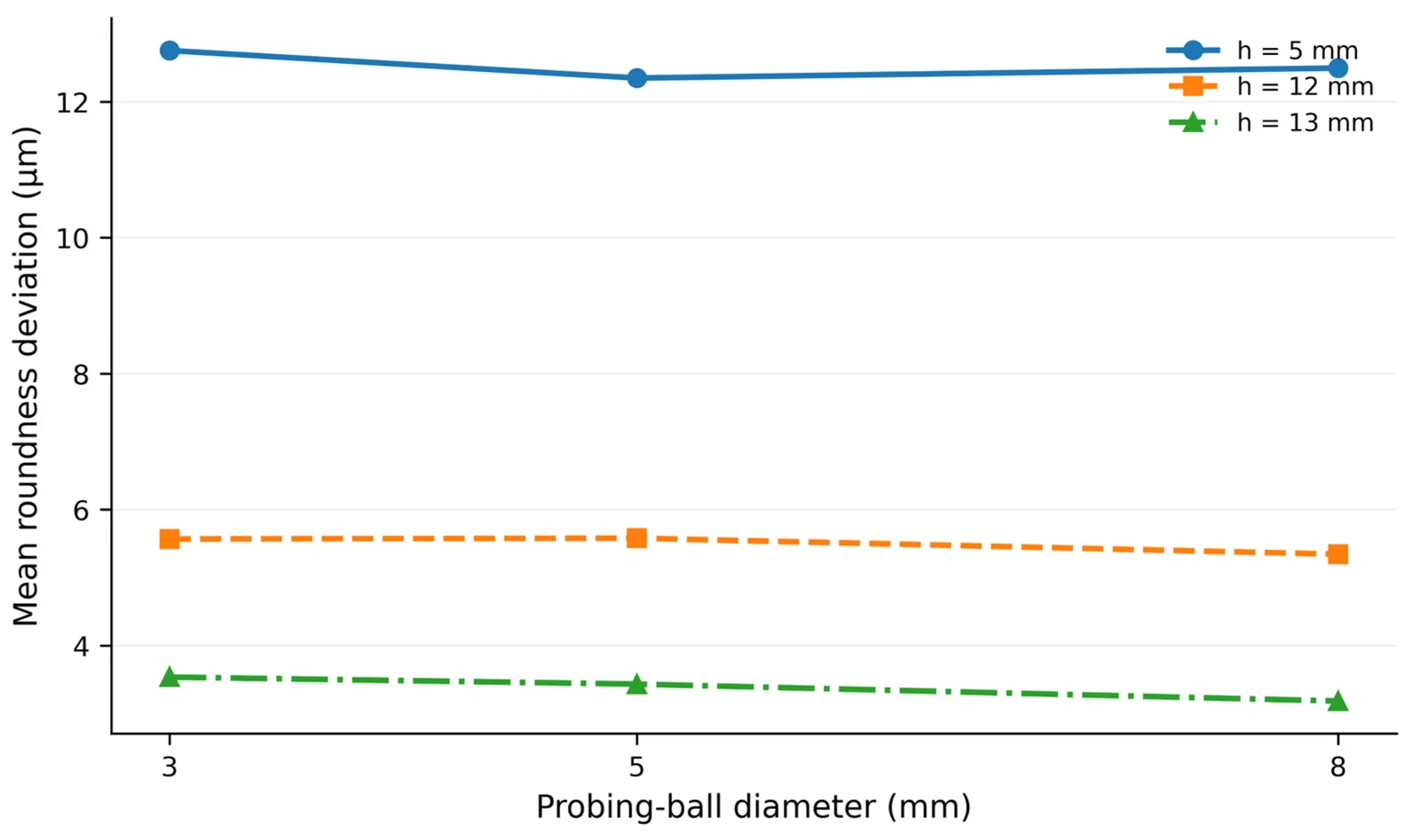
Mean roundness deviation of D19.8 as a function of probing-ball diameter at the three investigated axial positions.

**Figure 6:**
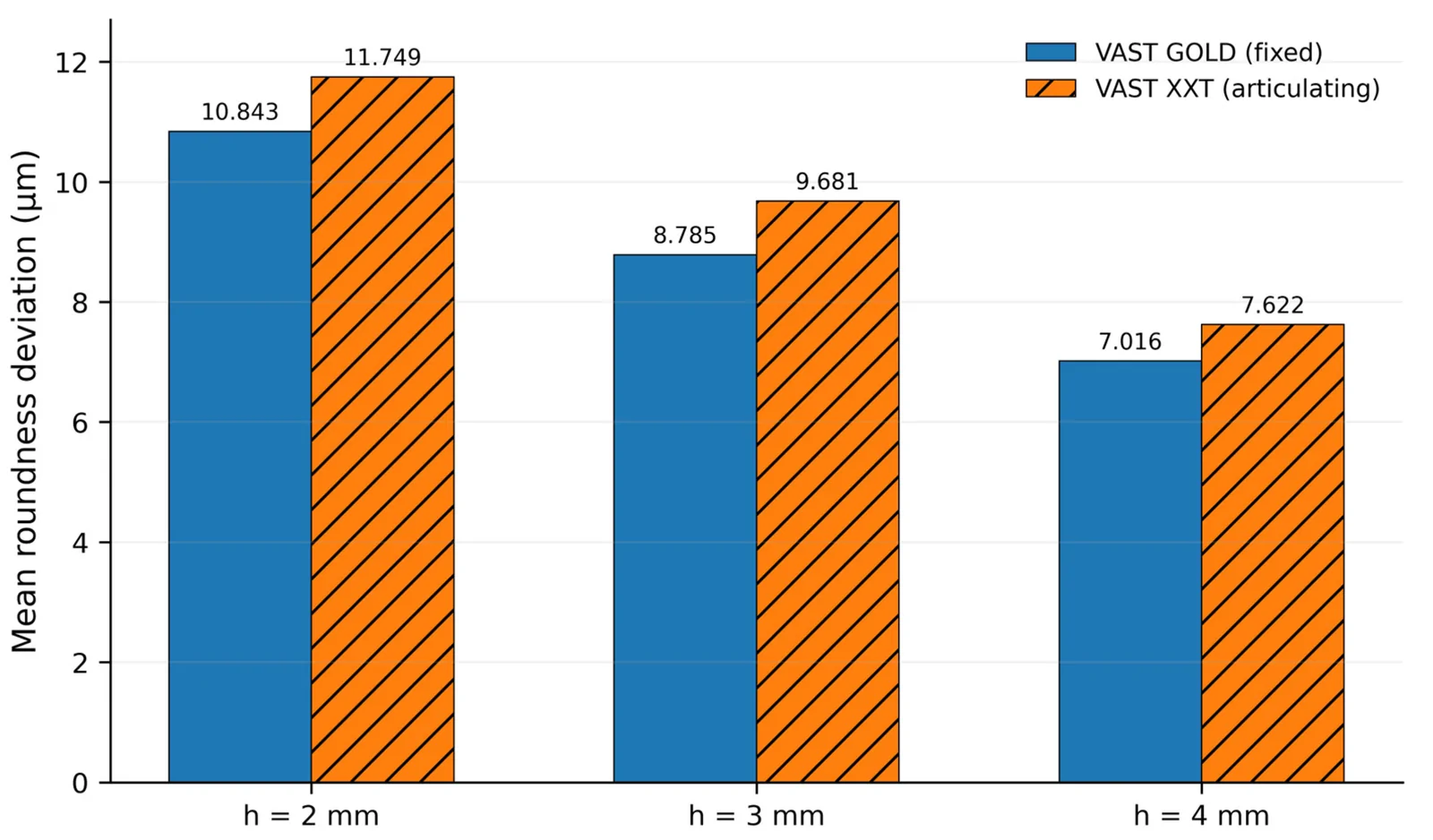
Mean roundness deviation obtained using the fixed VAST GOLD and articulating VAST XXT probing heads for D10.5 at h = 2, 3, and 4 mm.

The thesis provides more design detail than the summary article: its (Čása [14], Section 3.6) describes a planned three-factor, three-level space of 27 potential combinations but an implemented reduced set of 22 runs per feature for each probing head (44 observations per nominal diameter). Its Figure 3-6 (Čása [14]) show the 22-run configuration schedule, and Appendix A/B (Čása [14]) reports group sample counts and ANOVA tables. For example, D10.5 cylindricity lists N = 44, 16 model degrees of freedom and 27 residual degrees of freedom (Čása [14], Table 4-10/Appendix A-13); D6.5 cylindricity lists 28 residual degrees of freedom (Čása [14], Appendix A-2). These are model residual degrees of freedom, not repeated-setting pure-error degrees of freedom. Because 22 is less than 27, the assertion in the thesis that every three-factor combination was covered is inconsistent with its tabulated schedule. The manuscript treats this as a reduced, nonfully crossed design without inventing missing combinations or independent replication.

The documented design contains 22 configurations for each probing head and nominal diameter (44 measurement records per diameter), rather than a fully crossed 3 × 3 × 3 design with independently repeated cells. The three axial sections from a given measurement are not independent specimens. As a concrete illustration of the historical analysis, for D10.5 cylindricity, the reported model uses 44 records, model df = 16, residual df = 27 and F = 6.905; the reported factor-specific degrees of freedom are ball = 1, stylus = 11, points = 2 and head = 1 (Čása [14], Table 4-8; Appendix A-12). For D6.5 cylindricity, the reported residual df is 28 (Čása [14], Appendix A-2). Degrees of freedom differ with the nominal diameter and available stylus levels; their presence in historical ANOVA output does not demonstrate a valid pure-error estimate or independence. Interaction effects were not independently verified and the residual cannot be identified as within-configuration repeatability. Examples of the historical model structure are summarized in Table 5.

The original least significant difference (LSD) post hoc comparisons did not provide protection for the full family of tests across 24 datasets. No defensible multiplicity-adjusted pairwise *p*-values, effect-size estimates or confidence intervals can be recalculated from the summary output alone. In particular, nominal *p*-values near 0.05 should not be treated as robust evidence. Adjusted R^2^ indicates in-sample model fit, not valid factor separation, metrological accuracy or model-assumption compliance.

Descriptive within-level standard deviations and some 95% confidence intervals are reproduced in the thesis (Čása [14], Appendix A-1 and Table 4-9), but pooling different stylus configurations within a sampling level does not estimate within-configuration repeatability. Consequently, these intervals are descriptive for the observed design rather than independently validated uncertainty intervals. The thesis provides F-statistics and error degrees of freedom for the reported models, but its outputs do not establish residual independence or a valid error term for repeated probing of the same specimen.

In contrast with the unreplicated multifactor configurations, the separate ten-point screening used five repeated measurements per ball diameter and head, and D10.5 individual repeat values are tabulated in thesis (Čása [14], Tables 3-13 to 3-15 and 3-19 to 3-21). These repetitions allow description of within-screening variability only for those stated settings; they cannot be transferred as a pooled uncertainty budget or as residual error for the main 5/20/100-point study.

Additional multiplicity sensitivity analysis (new exploratory reanalysis of published summary *p*-values). A Holm step-down procedure was applied to the 24 historical omnibus *p*-values within each factor family (six cylindricity and 18 roundness strata), using *p* = 0.001 as a conservative proxy for entries reported as *p* < 0.001. The adjusted classifications remain conditional on the historical model and its unverified independence and variance assumptions. Holm correction addresses multiplicity, not confounding, pseudoreplication, residual misspecification or causal identifiability. Unadjusted LSD pairwise findings are reported as historical descriptions only.

## 3. Results

### 3.1. Sampling Density

Sampling density displayed the most consistent association in the historical exploratory model outputs. Figure 3 summarizes descriptive cylindricity means for the six nominal diameters. From 5 to 100 points per circular section, means increased from 7.050 to 10.097 µm (D6.5), 10.674 to 13.872 µm (D8.5), 8.686 to 13.830 µm (D10.5), 19.183 to 29.387 µm (D13), 3.510 to 12.761 µm (D16), and 9.850 to 14.084 µm (D19.8). The original ANOVA reported nominal *p* < 0.05 for the point-density term in each of the six cylindricity datasets (Table 6), but its design assumptions remain unverified. The largest descriptive absolute contrasts occurred for D13 (10.204 µm) and D16 (9.251 µm).

The numerical contrasts here describe differences in the reported evaluated form values for the available point sampling strategies. They do not indicate that the physical surfaces changed or, by themselves, demonstrate improved measurement accuracy. Denser sampling can reveal profile extrema missed by sparse sampling, but measurement error and the degree of convergence to a reference form operator were not established.

The same descriptive trend was seen for roundness in the available D19.8 results (Figure 4). At h = 5 mm, means at 5, 20 and 100 points were 9.880, 13.349 and 14.081 µm; at h = 12 mm, they were 4.633, 5.475 and 6.217 µm; and at h = 13 mm, 1.735, 3.832 and 4.387 µm. The original unadjusted LSD outputs labelled pairwise comparisons as nominally significant (*p* < 0.001), but these should be regarded as exploratory model-dependent findings rather than independently replicated differences.

### 3.2. Probing-Ball Diameter

The historical exploratory ANOVA suggested configuration-dependent associations for probing-ball diameter. For D19.8 roundness, the original model gave nominal *p*-values of 0.024602 at h = 5 mm (F = 4.24), 0.032676 at h = 12 mm (F = 3.88), and 0.206709 at h = 13 mm (F = 1.669). At h = 5 mm, the descriptive means for 3, 5 and 8 mm balls were 12.754, 12.349 and 12.496 µm, respectively. The unadjusted historical LSD outputs reported *p* < 0.001 for 3 versus 5 mm, *p* = 0.0057 for 3 versus 8 mm, and *p* = 0.095 for 5 versus 8 mm. At h = 12 mm, the corresponding means were 5.566, 5.578 and 5.342 µm; the historical LSD output gave *p* < 0.001 for 8 versus 3 mm and for 8 versus 5 mm, and *p* = 0.821 for 3 versus 5 mm. These pairwise *p*-values are retained only as exploratory, model-dependent descriptions: unverified independence, confounding and multiplicity preclude claims of confirmed differences. Figure 5 presents the descriptive means.

### 3.3. Stylus Shaft Length

In the historical exploratory ANOVA, stylus shaft length was the second most frequent factor to cross the nominal *p* < 0.05 threshold, with classifications dependent on feature diameter and axial position. For cylindricity, these were D6.5 (F = 4.975, *p* = 0.000370), D8.5 (F = 14.0423, *p* < 0.001), and D16 (F = 3.2068, *p* = 0.007103), or 3/6 datasets. For roundness, the historical nominal threshold was crossed in 8/18 datasets: D6.5 at h = 1.5 mm; D8.5 at h = 1.5, 3 and 4 mm; D10.5 at h = 4 mm; D16 at h = 3 and 5 mm; and D19.8 at h = 12 mm. The other ten datasets did not cross this threshold. These counts neither establish separately identifiable stylus effects nor permit a universal monotonic rule, because the original assumptions and factor separation have not been demonstrated.

### 3.4. Probing-Head Type

The historical exploratory summaries assigned fewer nominal *p* < 0.05 classifications to probing-head type than to sampling density. For D10.5 roundness (Figure 6), descriptive means with VAST GOLD and VAST XXT, respectively, were 10.843 and 11.749 µm at h = 2 mm (historical *p* = 0.256207), 8.785 and 9.681 µm at h = 3 mm (*p* = 0.047481), and 7.016 and 7.622 µm at h = 4 mm (*p* = 0.015385). The historical head-type term crossed the nominal 0.05 threshold in 3/18 roundness strata and in none of the six cylindricity strata. These model-dependent classifications cannot establish a universal head-type advantage or independent causal effect.

### 3.5. Preliminary Comparison with the Reference Instrument

The original thesis presented a 10-point preliminary comparison and summarized fractions of results within a historical ±0.001 mm screening threshold (roundness: 94.4% VAST GOLD and 86.1% VAST XXT; diameter: 97.2% and 83.3%). Its corresponding cylindricity summary stated 100% and 83.3%, respectively, but the underlying fixed-head cylindricity values contradict a 100% within-threshold interpretation. These fractions are reproduced solely to document the historical thesis report, not as independently validated reference-agreement rates. The multifactor analysis addresses a different set of configurations and a different sample count.

These within-threshold percentages omit signed differences and their dispersion. Hence, they cannot estimate bias, limits of agreement, expanded uncertainty or relative probing-head accuracy. They are retained solely as historical descriptive results of the restricted screening exercise. The separately reported Talyrond uncertainty of 0.1 µm is not sufficient to establish uncertainty-normalized agreement without the CMM contribution and full measurement conditions.

A further internal-consistency problem is visible in the thesis: its fixed-head cylindricity table reports absolute differences against Talyrond of 6.18 µm for D16, 15.74 µm for D8.5 and 4.69 µm for D6.5, although its accompanying prose calls 100% of cases within ±1 µm (Čása [14], Section 4.2.2, Table 4-5). The historical 100% percentage is therefore not independently supported by that source table and must not be construed as a quantitative accuracy or reference-agreement claim. The present manuscript does not endorse that historical percentage.

A direct numerical check of the six medium-ball screening values in the source thesis (Čása [14], Tables 4-2 and 4-5) gives signed CMM minus Talyrond differences in the rounded, selected medium-ball cylindricity values in micrometres: fixed VAST GOLD [−4.69, −15.74, −1.04, −4.36, −7.18, −0.91] and articulating VAST XXT [−5.09, −15.74, −0.04, −4.76, −5.18, −1.31], ordered by ascending feature diameter. The respective mean signed differences are −5.65 µm and −5.35 µm, and mean absolute differences are 5.65 µm and 5.35 µm (*n* = 6 configurations each). This comparison does not establish superiority of either head because the source reports rounded group values, a very limited selection of configurations, and not the operator/filter equivalence or task-specific uncertainty of the two instruments.

The screening comparison and the multifactor ANOVA answer different questions: selected ten-point outcomes compared descriptively with nominal reference values versus exploratory associations across factor configurations. Neither provides the paired uncertainty-normalized comparison needed to rank the probing heads by measurement accuracy. In particular, there is no justified conclusion that the fixed probing head is universally more accurate.

### 3.6. ANOVA Results Across Features

The historical exploratory ANOVA results are presented in two complementary tables. Table 6 reports F-statistics and nominal *p*-values for cylindricity at six nominal diameters. Table 7 reports nominal *p*-values for the eighteen roundness diameter–depth strata and identifies those below the unadjusted α = 0.05 screening threshold. These archived model classifications are not confirmatory tests. Selected F-statistics are discussed without implying independent causal effects.

All quoted F-statistics and nominal *p*-values reproduce the historical exploratory models and have not been independently recalculated. Large F-statistics may arise when between-level variation is high relative to the model residual mean square; although the archived outputs report residual degrees of freedom, they do not establish an independently replicated pure-error estimate or validate the residual assumptions. The original post hoc comparisons were not multiplicity-corrected, so the configuration-specific borderline findings in particular require caution.

For cylindricity (Table 6), the historical exploratory ANOVA assigned nominal *p* < 0.001 to the sampling-density term in all six feature-specific models (F = 44.98 for D13 to F = 1227.74 for D19.8). Stylus shaft length crossed nominal *p* < 0.05 for D6.5 (F = 4.975, *p* < 0.001), D8.5 (F = 14.042, *p* < 0.001), and D16 (F = 3.207, *p* = 0.007); probing-ball diameter did so only for D19.8 (F = 4.41, *p* = 0.022), whereas head type did not cross the nominal threshold in any of the six models. The pattern describes the fitted historical data but does not isolate independent effects because factor dependence, absent exact-configuration replication and model assumptions remain unresolved.

The historical least significant difference (LSD) post hoc outputs provide only an exploratory description of nominal pairwise contrasts. For D6.5 cylindricity, the archived pairwise outputs reported *p* < 0.001 for 5 versus 20 and 5 versus 100 points and *p* = 0.00072 for 20 versus 100 points. For D19.8, they reported *p* < 0.001 for each sampling-density pair. For D19.8 ball diameter, archived unadjusted values were *p* < 0.001 for 3 versus 5 mm, *p* = 0.0065 for 3 versus 8 mm and *p* = 0.0875 for 5 versus 8 mm. These are not confirmed between-configuration differences: the LSD comparisons were not familywise adjusted and inherit the historical model’s unverified independence, residual and factor-separation assumptions. Adjusted R^2^ ranged from 0.687 to 0.985 for the cylindricity fits (highest for D19.8, 0.984952), but this represents only in-sample fit and does not validate those assumptions.

The historical roundness ANOVA summaries show a consistent descriptive pattern across the six feature diameters and eighteen axial-section strata. In Table 7, the archived models assigned nominal *p* < 0.05 to the point-density term in 18/18 strata; these values do not constitute confirmatory significance because the sampling units are not independent. For D19.8 at h = 5 mm, mean roundness was 0.009880, 0.013349 and 0.014081 mm at 5, 20 and 100 points, respectively. The historical unadjusted LSD outputs gave nominal *p* < 0.001 for all three pairs. These values are presented solely to describe the original exploratory model and must not be interpreted as statistically confirmed independent differences. A similar pattern in archived model outputs occurred across the other roundness strata.

In the historical unadjusted exploratory roundness ANOVAs, the stylus-length term crossed nominal *p* < 0.05 in 8/18 strata: D6.5 at h = 1.5 mm; D8.5 at h = 1.5, 3 and 4 mm; D10.5 at h = 4 mm; D16 at h = 3 and 5 mm; and D19.8 at h = 12 mm. The ball-diameter term crossed that threshold in 3/18 strata (D8.5 at h = 4 mm; D19.8 at h = 5 and 12 mm), and the head-type term in 3/18 (D8.5 at h = 4 mm; D10.5 at h = 3 and 4 mm). These are counts of nominal historical classifications, not established configuration-specific causal effects. Table 7 therefore documents heterogeneity of exploratory associations rather than proving factor independence.

Selected historical F-statistics provide context for the exploratory classifications in Table 7. The archived point-density F-values included 1196.21 (D19.8, h = 5 mm), 686.994 (D19.8, h = 13 mm), 553.164 (D8.5, h = 3 mm) and 536.620 (D16, h = 5 mm), with nominal *p* < 0.001. For stylus length, the largest reported F was 19.425 (D8.5, h = 4 mm), and for head type at D10.5, the reported F-values were 4.312 (h = 3 mm, *p* = 0.047) and 6.693 (h = 4 mm, *p* = 0.015). Their magnitude cannot overcome uncertainty about independence, residual variation and confounding; they should be read as archived model outputs, not confirmatory evidence.

**Figure 7:**
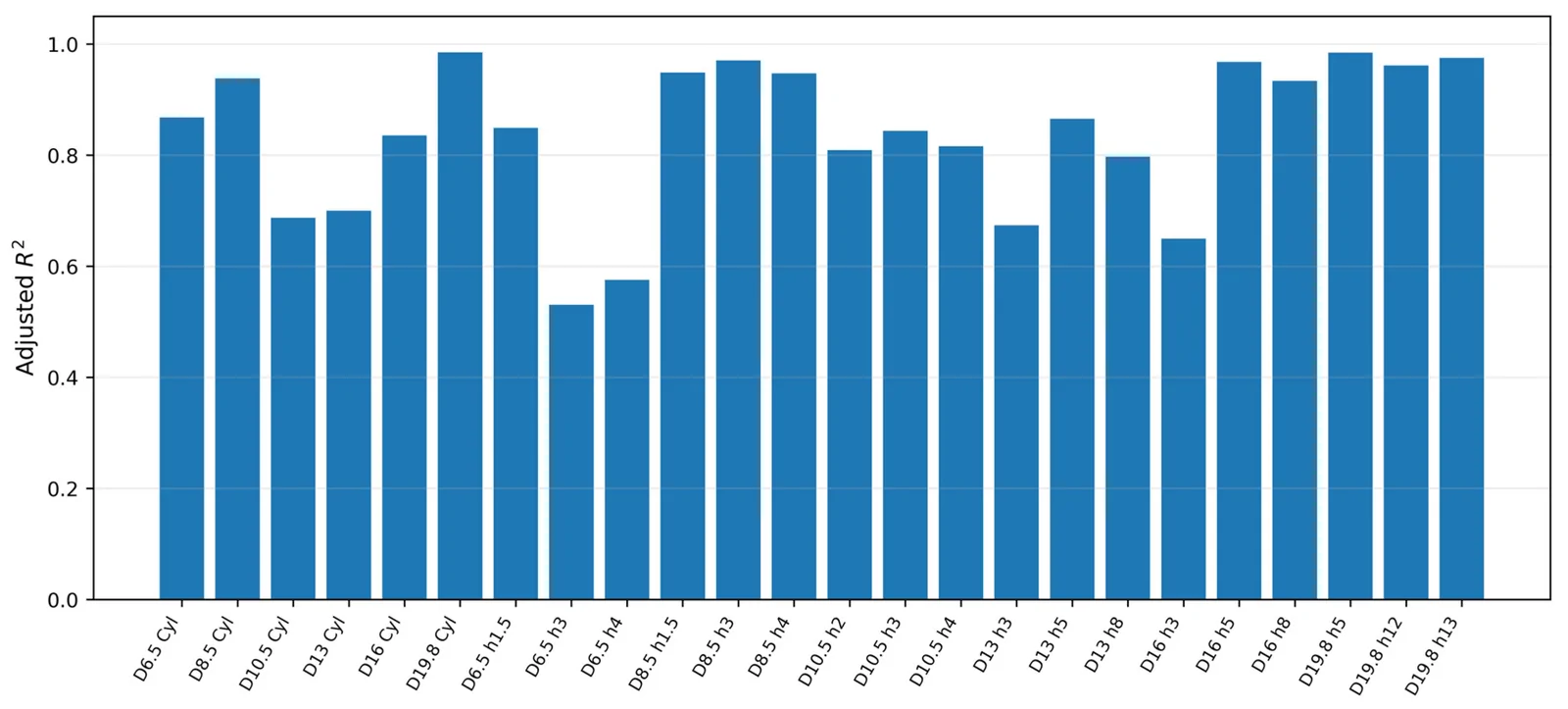
Adjusted R^2^ values of the ANOVA models for the six cylindricity datasets and 18 roundness datasets. All values are reproduced from the statistical outputs reported in the diploma thesis.

Model fit varied among the individual geometries (Figure 7). Adjusted R^2^ ranged from 0.687 to 0.985 for cylindricity and from 0.530 to 0.985 for roundness. The highest values were obtained for D19.8 cylindricity (0.984952), D19.8 roundness at h = 5 mm (0.984570), and D19.8 roundness at h = 13 mm (0.974992). Conversely, lower adjusted R^2^ values for several smaller-diameter or selected-depth datasets indicate that a larger share of their variability remained outside the fitted main-factor model. These in-sample fit measures do not validate model assumptions, independence, nominal significance classifications or causal interpretations.

Together, Table 6 and Table 7 reproduce historical exploratory results in which nominal *p* < 0.05 was reported for the sampling-density term in 24 of 24 strata. Associations for stylus length, ball diameter and probing-head type were less consistently reported. None of these counts establish independent causal influence because model assumptions, within-feature dependence and factor separation remain unresolved.

Sensitivity to multiple testing: After Holm adjustment of the historical omnibus *p*-values within each factor family, 24/24 sampling-density, 9/24 stylus-length, 0/24 ball-diameter and 0/24 probing-head results remained below 0.05. These are exploratory robustness classifications for the recorded *p*-values, not confirmatory evidence; rounding, dependence and model specification affect their interpretation.

## 4. Discussion

Across the descriptive averages, increasing sample density is associated with higher reported form deviations in all six cylindricity series. This is consistent with better detection of extrema by a denser discrete representation; it is not proof that the part physically changed or that measurement accuracy increased. The analysis extends the thesis by placing all six diameter-dependent changes on a common absolute and relative scale (Table 8) and by explicitly distinguishing persistent sampling associations from less consistent probe-configuration findings.

Here “dominant” means the most consistent association in the supplied exploratory ANOVA tables, not a demonstrated independent causal effect or evidence of superior accuracy. The direction and magnitude of a measured difference depend on the spatially sampled surface, fitting operator, probe accessibility, stylus mechanics and possible feature-specific correlations. The exact CALYPSO circle and cylinder fitting operators, filtering and probe compensation settings cannot be independently confirmed from the supplied record, precluding a mechanistic decomposition of those influences.

Table 8 provides a quantitative cross-feature comparison calculated directly from the reported mean values: changes between 5 and 100 points per circular section range from 3.047 to 10.204 µm in absolute terms and differ considerably as percentages of the five-point mean. These are observed contrasts under the historical, unbalanced sampling design, not controlled causal effect estimates. In particular, relative percentages for low baseline deviations can be large without implying a corresponding gain in accuracy. This comparison is a new descriptive synthesis, not a new inferential test.

The original contribution is not the already expected observation that more sampled points can detect previously missed profile extrema. Rather, this study offers a documented, case-specific secondary analysis: comparable absolute and relative contrasts across six particular drilled features, sensitivity checks of historical statistical classifications, transparent access to individual exported observations and identification of evaluation and reference-comparison limitations. These are descriptive and methodological contributions tied to the investigated instrument, specimens and configurations. They are not evidence of a new physical mechanism, a universal sampling threshold or performance across a broader population of CMM measurement tasks.

The historical exploratory analysis suggested a non-universal association with stylus length. This is compatible with previous observations that probe geometry, orientation, and stylus length can influence CMM results [7,8,9]. In the historical unadjusted ANOVA outputs, the stylus-length term had nominal *p* < 0.05 in 3 of 6 cylindricity datasets and 8 of 18 roundness datasets; these model-dependent counts do not establish independent-factor effects. Importantly, the data do not support a simple monotonic rule that increasing stylus length necessarily increases the evaluated form deviation. The comparison with earlier probe-focused studies therefore supports a task-specific interpretation: stylus configuration should be validated for the actual feature diameter, accessibility, and measurement depth rather than treated as an independent universal correction factor.

The historical unadjusted ANOVA indicated nominal *p* < 0.05 for probing-ball diameter only in selected configurations, particularly for D19.8; no probing-ball tests retained that classification in the across-stratum Holm sensitivity screening. Because the experiment did not include independent surface-topography characterization, the observed differences should not be attributed uniquely to mechanical filtering of roughness. They are more conservatively interpreted as a probe-configuration dependence of the evaluated form result.

The historical preliminary screening reported different fractions within its ±0.001 mm criterion for fixed and articulating systems, but these fractions cannot establish relative accuracy. In particular, the source thesis reports a 100% fixed-head cylindricity success rate despite listing multiple reference differences exceeding the criterion. Because the count and calculation are inconsistent, the reported percentage is not retained as validated evidence. The multifactor results address a separate exploratory statistical question and do not support declaring one head universally superior.

Generalisability is limited by the use of one CMM, two ZEISS probing configurations, six drilled cylindrical features and three discrete-point sampling levels. The same physical features were measured under several configurations, exact combinations were not independently replicated, factor settings were partly dependent and the geometrical evaluation operators were not demonstrated to be equivalent to those of the reference instrument. Task-specific combined measurement uncertainty was not established. Therefore, neither historical nominal statistical findings nor exploratory sensitivity checks should be extrapolated as independent causal or accuracy effects, universal stylus rules or validated inspection settings. Confirmatory research would require independently manufactured specimens, a replicated and suitably randomized design, consistently documented evaluation operators and a task-specific uncertainty assessment.

The measurement sequence and any randomization or blocking against temporal drift are not specified in the available report; the documented temperature range alone does not exclude thermal drift or order effects. Repeatability of individual fixed configurations cannot be separated from cross-configuration scatter in the supplied summaries. In addition, no complete task-specific uncertainty budget, confidence intervals or traceable agreement metrics are available. Priority future work should employ independently manufactured replicated features, a balanced crossed or estimable blocked design, recorded order and environmental history, residual and correlation diagnostics, multiplicity-controlled inference, an uncertainty budget, and an explicit discrete-point versus scanning comparison.

For industrial application, these results should be treated as a case study illustrating why point density and probing configuration deserve task-specific verification, not as an empirically validated ranking of measurement settings. The observed differences in evaluated form values do not demonstrate improved accuracy, and the reference comparison does not establish operator equivalence or combined measurement uncertainty. Any selection of probing head, ball diameter, stylus length or point count for another part or tolerance must therefore be justified by independent validation under that particular measurement task.

A proposed validation workflow—rather than a recommendation experimentally established by the present data—is to specify the required form tolerance and measurement decision rule, document the geometric evaluation operator, choose an initial sampling pattern compatible with feature accessibility, and compare it with successively denser strategies on independently repeated parts. Measurement time, environmental conditions, stylus reach and a task-specific uncertainty budget should then inform the selected strategy. No numerical minimum point count, acceptance threshold or general industrial optimum can be inferred from this six-feature dataset.

The reported descriptive values can be compared directly in physical units: D13 mean cylindricity rises by 10.204 µm between 5 and 100 points per section; D19.8 roundness at h = 5 mm increases by 4.201 µm. However, absent a stated production form tolerance, these are observed contrasts rather than pass/fail or capability conclusions.

### Reanalysis of the Machine-Readable Data Export

A feature-level export comprising individual CMM-reported geometric characteristics from the fixed and articulating heads, along with a separate repeated 10-point preliminary series and Talyrond reference values, was recovered and curated. The supporting workbook lists the original source labels, nominal feature size, evaluated characteristic, axial depth, head, probing configuration and result, where identified unambiguously.

For the main multifactor export, 24 stratified Kruskal–Wallis comparisons (six cylindricity and eighteen roundness strata) contrasted reported form values across sampling levels. The calculated Holm-adjusted *p*-values were below 0.05 in all 24 comparisons under the nominal test assumptions. However, the same physical features were measured repeatedly, so the Kruskal–Wallis independence assumption is not met. These results are solely exploratory sensitivity descriptions of rank separation, not an independent validation of ANOVA, confirmatory hypothesis tests or evidence of a causal effect. Independently manufactured specimens with independent assignment or an appropriate repeated-measures design would be needed for population-level inference; neither is available here.

The separate 10-point experiment provides actual within-configuration repeats; repeatability standard deviations in the supplementary workbook refer only to these repeated preliminary measurements and must not be transferred to the unreplicated multifactor configurations.

Observation-level differences between CMM and Talyrond results are presented only where corresponding measured characteristics and physical axial positions can be reasonably identified. Sampling densities and evaluation settings may differ between instruments, and the Talyrond expanded uncertainty U = 0.1 µm (k = 2) is not a combined task-specific uncertainty of inter-instrument agreement. For the third D19.8 roundness section, the CMM export uses h13 and the Talyrond export h15; their physical coincidence is not documented, so this pair is excluded from depth-matched reference agreement. Separately measured CMM diameter results bearing h17 are not used to infer the roundness section depth.

Data availability: Machine-readable individual exported feature results, a data dictionary, preliminary repeated measurements, source-indexed descriptive summaries and reference comparisons are provided as Supplementary Dataset S1. Original point clouds and complete software evaluation settings are not included. The original experimental work was documented in the 2025 diploma thesis [14].

## 5. Conclusions

The results describe systematic differences in the CMM-reported roundness and cylindricity values across the investigated sampling and probing configurations. The most consistent descriptive pattern in this dataset is that denser point sampling tends to report larger form deviations, while other probe-related associations are more feature-specific. This is a secondary exploratory analysis of a limited, unbalanced experiment on six physical features, and it does not establish independently causal effects, superior measurement accuracy, or a generally optimal CMM configuration. The main findings and their limitations are summarized below.

These conclusions concern the observed variation in evaluated form values, not a demonstrated increase in accuracy or a formally validated measurement capability. Statistical significance statements below are reproduced from the historical analysis and are conditional on unverified assumptions and unadjusted testing. Incomplete replication, possible dependence, unknown evaluation settings and the absence of a full uncertainty budget preclude universal causal or accuracy claims. Although the Talyrond expanded reference uncertainty is U = 0.1 µm (k = 2), a full combined uncertainty for the comparison remains unavailable.

The Holm screening of published summary statistics retains the sampling-density association but not the isolated ball-diameter and head-type effects. These adjusted counts remain conditional on the historical model structure and should not be interpreted as independent verification of the experimental inference.

1. In the historical exploratory ANOVA, the sampling-density term met the nominal *p* < 0.05 threshold in 6/6 cylindricity and 18/18 roundness strata, and those classifications persisted in the Holm sensitivity screening. Because independence and error-model assumptions are not established, these results should not be described as confirmatory statistical significance or independent causal evidence.
2. Increasing point density generally increased the evaluated form deviation. Representative changes were 7.050 to 10.097 µm for D6.5 cylindricity and 9.880 to 14.081 µm for D19.8 roundness at h = 5 mm.
3. Historical exploratory ANOVA identified nominal *p* < 0.05 for stylus shaft length in 3/6 cylindricity and 8/18 roundness strata; nine of the 24 tests retained this classification under the Holm sensitivity calculation. These results are conditional associations, not separately identified causal effects.
4. Historical exploratory *p*-values for ball diameter fell below 0.05 in 1/6 cylindricity and 3/18 roundness strata, but none survived the across-stratum Holm screening. Original unadjusted pairwise LSD results remain descriptive and vulnerable to multiplicity and confounding.
5. Historical exploratory tests did not identify nominal *p* < 0.05 for head type in cylindricity and did so in only 3/18 roundness strata; none survived the across-stratum Holm screening. The separate 10-point screening cannot establish a head-accuracy ranking, particularly given an inconsistency in its original cylindricity summary.
6. The newly consolidated absolute and relative comparison shows variability among the six measured feature diameters: between 5 and 100 points per section, mean cylindricity contrasts range from 3.047 to 10.204 µm (Table 8). These case-specific descriptive contrasts cannot be generalized to other features or interpreted as improvements in accuracy or as independent causal effects.
7. Within the configurations examined, the instrument specification alone is insufficient to characterize task-specific CMM form-measurement performance. Verification requires a controlled geometrical evaluation operator, suitably documented sampling and probing procedures, independently replicated specimens and an uncertainty budget; these were not all available in the source experiment.

Future work should extend the experiment toward a complete task-specific uncertainty budget, interaction analysis, independent surface-topography characterization, and direct comparison between discrete-point and scanning strategies.

## Figures and Tables

**Table 1:** Reference cylindricity measured using the Taylor Hobson Talyrond 585 LT.

Nominal Diameter (mm)	Reference Cylindricity (mm)
6.5	0.01309
8.5	0.02774
10.5	0.01104
13	0.02836
16	0.01318
19.8	0.01391

**Table 2:** Descriptive ten-point screening comparison of one medium-ball configuration per diameter, transcribed from (Čása [14], Tables 4-2 and 4-5). CMM values are rounded in the source; the comparisons are not uncertainty-normalized.

D (mm)	Talyrond (µm)	VAST GOLD, Medium Ball (µm)	VAST XXT, Medium Ball (µm)
6.5	13.09	8.40	8.00
8.5	27.74	12.00	12.00
10.5	11.04	10.00	11.00
13	28.36	24.00	23.60
16	13.18	6.00	8.00
19.8	13.91	13.00	12.60

**Table 3:** Axial positions used for roundness measurement and for constructing the three-section cylindricity evaluation.

Nominal Diameter (mm)	Section 1 (mm)	Section 2 (mm)	Section 3 (mm)
6.5	1.5	3	4
8.5	1.5	3	4
10.5	2	3	4
13	3	5	8
16	3	5	8
19.8	5	12	13

**Table 4:** Probing-ball diameters used for each nominal cylindrical feature.

Nominal Diameter (mm)	Small Ball (mm)	Medium Ball (mm)	Large Ball (mm)
6.5	1	1.5	2
8.5	1.5	2	3
10.5	1.5	2	3
13	2	3	5
16	2	3	5
19.8	3	5	8

**Table 5:** Source-documented examples of historical model structure (Čása [14], Table 4-8 and Appendix A). These are residual model degrees of freedom, not independently replicated pure-error degrees of freedom.

Outcome	Observations	Model df	Residual df
D10.5 cylindricity	44	16	27
D6.5 cylindricity	44	15	28

**Table 6:** Historical exploratory ANOVA results for cylindricity.

D (mm)	Ball F	Ball *p*	Stylus F	Stylus *p*	Points F	Points *p*	Head F	Head *p*
6.5	0.005	0.994857	4.975	0.000370 *	97.780	<0.001 *	0.052	0.820628
8.5	0.770	0.473251	14.042	<0.001 *	231.936	<0.001 *	0.462	0.502875
10.5	0.009	0.924485	0.466	0.908462	45.638	<0.001 *	0.587	0.450344
13	0.079	0.780362	0.893	0.551018	44.976	<0.001 *	0.810	0.375475
16	0.058	0.811208	3.207	0.007103*	77.913	<0.001 *	0.236	0.631285
19.8	4.410	0.021648 *	1.230	0.314106	1227.740	<0.001 *	0.100	0.749978
Significant datasets, *n*/*N*	1/6		3/6		6/6		0/6	

Note: * denotes a nominal unadjusted *p*-value below 0.05 under the original model, not a confirmatory finding; the final row counts such nominal classifications.

**Table 7:** Historical exploratory ANOVA nominal *p*-values for roundness by nominal diameter and axial position. Bold values denote unadjusted *p* < 0.05 under the original model, not confirmed independent effects; the final row counts such nominal classifications.

D (mm)	h (mm)	Ball Diameter, *p*	Stylus Length, *p*	Point Density, *p*	Head Type, *p*
6.5	1.5	0.608	0.002	<0.001	0.708
6.5	3	0.970	0.144	<0.001	0.691
6.5	4	0.987	0.709	<0.001	0.852
8.5	1.5	0.664	<0.001	<0.001	0.290
8.5	3	0.180	<0.001	<0.001	0.648
8.5	4	0.030	<0.001	<0.001	0.038
10.5	2	0.959	0.921	<0.001	0.256
10.5	3	0.579	0.148	<0.001	0.047
10.5	4	0.499	<0.001	<0.001	0.015
13	3	0.790	0.565	<0.001	0.403
13	5	0.456	0.585	<0.001	0.152
13	8	0.084	0.547	<0.001	0.074
16	3	0.895	0.044	<0.001	0.980
16	5	0.751	0.003	<0.001	0.587
16	8	0.612	0.0504	<0.001	0.576
19.8	5	0.025	0.317	<0.001	0.751
19.8	12	0.033	<0.001	<0.001	0.949
19.8	13	0.207	0.145	<0.001	0.580
Significant datasets	*n*/*N*	3/18	8/18	18/18	3/18

Note: *p*-values are shown to three decimal places, except the borderline value 0.0504; values below 0.001 are reported as *p* < 0.001. Bold values indicate *p* < 0.05.

**Table 8:** Newly consolidated descriptive contrast in mean cylindricity between five and 100 points per circular section (three sections per cylinder; totals of 15 and 300 sampled points). Values are derived from previously published group means, without new measurements or hypothesis tests.

D (mm)	5 pts (µm)	100 pts (µm)	Difference (µm)	Relative Change (%)
6.5	7.050	10.097	3.047	43.2
8.5	10.674	13.872	3.198	30.0
10.5	8.686	13.830	5.144	59.2
13	19.183	29.387	10.204	53.2
16	3.510	12.761	9.251	263.6
19.8	9.850	14.084	4.234	43.0

## Data Availability

The original contributions presented in this study are included in the article/Supplementary Material. Further inquiries can be directed to the corresponding author.

## Supplementary Materials

The following supporting information can be downloaded at: https://www.mdpi.com/article/10.3390/ma19214721/s1, Supplementary Dataset S1 contains the machine-readable experimental results, configuration records, reference measurements, and exploratory reanalysis tables described in this article. The file is supplied separately with the revised submission.

## References

[1] Hocken R.J., Pereira P.H. (2011). Coordinate Measuring Machines and Systems.

[2] JCGM (2012). International Vocabulary of Metrology—Basic and General Concepts and Associated Terms (VIM).

[3] National Physical Laboratory (2004). Good Practice Guide No. 43—CMM Probing.

[4] (2011). Geometrical Product Specifications (GPS)—Cylindricity—Part 1: Vocabulary and Parameters of Cylindricity.

[5] (2011). Geometrical Product Specifications (GPS)—Cylindricity—Part 2: Specification Operators.

[6] (2020). Geometrical Product Specifications (GPS)—Acceptance and Reverification Tests for Coordinate Measuring Systems (CMS)—Part 5: Coordinate Measuring Machines (CMMs) Using Single and Multiple Stylus Contacting Probing Systems Using Discrete Point and/or Scanning Measuring Mode.

[7] Ali S.H.R. (2010). Probing system characteristics in coordinate metrology. Meas. Sci. Rev..

[8] Mazur T., Szymanski T., Samociuk W., Rucki M., Ryba T. (2025). The impact of probe angle and swivel length on contact point identification in coordinate measuring machine measurements: A case study. Sensors.

[9] Parthiban M., Harinath M., Kirubakaran M. (2021). Measurement of roundness on the bearing by varying probe length in CMM. Mater. Today Proc..

[10] Stępień K., Kmiecik-Sołtysiak U., Čepová L., Zuperl U. (2024). Problems of measurements of roundness deviations with the use of coordinate measuring machines. MM Sci. J..

[11] Liu F., Cao Y., Li T., Ren L., Zhi J., Yang J., Jiang X. (2023). An iterative minimum zone algorithm for assessing cylindricity deviation. Measurement.

[12] Štrbac B., Ranisavljev M., Orošnjak M., Havrlišan S., Dudić B., Savković B. (2024). Unsupervised machine learning application in the selection of measurement strategy on coordinate measuring machine. Adv. Prod. Eng. Manag..

[13] Sato O., Takatsuji T., Matsuzaki K., Watanabe M., Kajima M., Miura Y., Nakanishi S. (2024). Practical experimental design and uncertainty evaluation method for dimensional and form measurements using coordinate measuring machines. Measurement.

[14] Čása J. (2025). Analýza Procesu Měření Tvarových Prvků, n.a CMM. Diploma Thesis.

